# Predicting Bacteriophage Enzymes and Hydrolases by Using Combined Features

**DOI:** 10.3389/fbioe.2020.00183

**Published:** 2020-03-24

**Authors:** Hong-Fei Li, Xian-Fang Wang, Hua Tang

**Affiliations:** ^1^Department of Pathophysiology, Key Laboratory of Medical Electrophysiology, Ministry of Education, Southwest Medical University, Luzhou, China; ^2^School of Computer and Information Engineering, Henan Normal University, Henan, China

**Keywords:** bacteriophage enzymes, hydrolase, analysis of variance, sequence feature, classification

## Abstract

Bacteriophage is a type of virus that could infect the host bacteria. They have been applied in the treatment of pathogenic bacterial infection. Phage enzymes and hydrolases play the most important role in the destruction of bacterial cells. Correctly identifying the hydrolases coded by phage is not only beneficial to their function study, but also conducive to antibacteria drug discovery. Thus, this work aims to recognize the enzymes and hydrolases in phage. A combination of different features was used to represent samples of phage and hydrolase. A feature selection technique called analysis of variance was developed to optimize features. The classification was performed by using support vector machine (SVM). The prediction process includes two steps. The first step is to identify phage enzymes. The second step is to determine whether a phage enzyme is hydrolase or not. The jackknife cross-validated results showed that our method could produce overall accuracies of 85.1 and 94.3%, respectively, for the two predictions, demonstrating that the proposed method is promising.

## Introduction

Bacteriophage, as safe agent, can lyse and infect specific bacteria without destroying natural beneficial microflora (Parmar et al., 2018). Hydrolytic enzymes encoded by phages are key ingredients of lysis, which is helpful to fighting bacterial pathogens, especially those that cannot be killed by antibiotics and chemicals. In fact, in some countries, they have been used therapeutically to treat bacterial infections that do not respond to antibiotics (Thiel, 2004; Parfitt, 2005; Keen, 2012). They have also been used as a food safety tool to reduce bacterial contamination (Pirisi, 2000). Hence, rapid detection of bacteriophage and hydrolase responsible for antibacterial drugs is a growing necessity for public health.

Because of abuse of antibiotics, certain resistant viruses cannot be effectively controlled. This problem can be resolved by therapy of phage hydrolytic that disintegrates host viruses during releasing progeny phage. Therefore, the identification of hydrolases encoded by phages has become an important research topic. It not only has been studied in chemistry and physics through experimental methods, but also achieved good results in theory through recently popular machine learning algorithms. Some experiments have been performed to study the function of phage hydrolase (Kimura and Itoh, 2003; Rodriguez-Rubio et al., 2013). In addition, in the study of host cell lysis by hydrolytic enzyme activation, Kovalenko et al. (2019) found that the calcium could regulate phage-induced bacterial lysis. Although those biochemical-based methods can accurately recognize phage hydrolases and clearly elucidate the functional mechanism of the enzyme, it is time-consuming and expensive. Additionally, biochemical experiments always need rigorous experimental conditions, which will prevent most of scholars from doing more in-depth studies. Computational methods provide another chance to study phage hydrolase without the disadvantage of biochemical-based methods. Phylogenetic analysis or similarity search could find relative conservation of motifs among related species (Lin and Li, 2011; Liu et al., 2019). However, it is extremely diverse for phage Open Reading Frames (ORFs), of which more than 70% of them cannot find out similar genes with annotated functions in GenBank (Seguritan et al., 2012). Moreover, it is also time-consuming.

With the accumulation of more and more postgenomic data, some computational methods have been proposed to study the function of phage proteins. Riede and his colleagues (Riede et al., 1987) have proposed a model to predict tail-fiber proteins’ three-dimensional structure of T-even–type phages. The results are consistent with electron microscopic data. Subsequently, a computer program was developed to identify DNA-binding regulatory proteins in bacteriophage T7 (White, 1987; Song et al., 2014; Zou et al., 2016a; Qu et al., 2019). Recently, the virion proteins encoded by phages were studied by using naive Bayes combined with primary sequence information (Feng et al., 2013). The proposed model could yield the overall accuracy (Ac) of 79.15%. By using feature selection technique, the overall Ac was improved to 85.02% (Ding et al., 2014). A free webserver called PVPred (Ding et al., 2014) was constructed for predicting phage virion proteins.

The success of previous works on the prediction of phage functional proteins (Feng et al., 2013; Ding et al., 2014) and enzyme prediction (Zuo et al., 2014; Ding H. et al., 2016) provided good strategy to discriminate hydrolases encoded by phages by transforming protein sequences into digital features and further establishing machine learning-based models. Thus, this work aims to develop a powerful computational model to recognize phage hydrolase by combining feature selection and expression of multiple features. The entire experiment was divided into two steps. First is to discriminate phage enzymes from phage nonenzymes and then to identify phage hydrolases from phage enzymes. In this model, the support vector machine (SVM) was applied as the algorithm to perform the classification. Different features were proposed to formulate protein samples and then inputted into SVM. The best features that can achieve the maximum accuracies were discovered by using analysis of variance (ANOVA). The model’s performance was estimated by using jackknife cross-validation.

## Materials and Methods

### Benchmark Dataset

Constructing a reliable benchmark dataset could guarantee the reliability of the proposed computational model (Ma et al., 2014; Liang et al., 2017; Yang et al., 2017; Wang et al., 2018; Cheng et al., 2019; Hu et al., 2019; Zheng et al., 2019). In this work, samples were gained from Ding H. et al. (2016), which were rigorously screened through the following three steps: (1) phage proteins have been annotated by standard operating procedure for UniProt manual curation (Swiss-Prot); (2) protein sequences samples containing illegal characters were deleted; (3) sequence identity in the dataset must be less than 30%, which was implemented by CD-HIT (Fu et al., 2012) software. Consequently, the definitive benchmark dataset contains 255 phage proteins, of which 124 proteins belong to phage enzymes (positive samples of set 1), and the remaining 131 are phage nonenzymes (negative samples of set 1). Furthermore, 124 phage enzymes are divided into 69 hydrolases (positive samples of set 2) and 55 nonhydrolases (negative samples of set 2), respectively. The following calculations are all based on these data.

### Protein Feature Extraction

The perfect expression of protein sequences by digital features can dramatically increase the Ac and robust of computing models (Wang et al., 2008, 2010; Song et al., 2010, 2018; Zuo et al., 2017; Basith et al., 2018; Chen W. et al., 2018; Wei et al., 2018b; Boopathi et al., 2019; Ding et al., 2019; Manavalan et al., 2019b; Shen et al., 2019; Tan et al., 2019; Zhang and Liu, 2019; Zhu et al., 2019). The specific order of residues in the peptide sequence dictates the protein to fold up into a special three-dimensional structure. Thus, the interaction between two residues in a protein is a main factor to characterize the protein. In the past 20 years, scholars have developed dipeptide composition to formulate peptide samples (Tang et al., 2016). However, the feature can only describe the short-range interaction between two residues. In fact, there are lots of long-range interaction for a protein in three-dimensional space. For example, the secondary structures (α helix and β sheet) were formed by the interaction of two nonadjoining residues. Hence, it will be more reasonable to investigate the performance of other kinds of correlations.

Based on the above analysis and other peer works (Ding and Li, 2015), in this work, the g-gap dipeptide composition (GGDC), which is extended from general dipeptide composition, is used as the main feature to denote the residues’ correlation in the original peptide sequence. For the perfect expression of the sample, the combination of GGDC, pseudo–amino acid composition (PseAAC), grouped tripeptide composition (GTPC), and composition transition and distribution (CTD) is used as the final feature vector. Pseudo–amino acid composition provides the correlation of physical and chemical properties between two residues (Chen et al., 2016; Yang et al., 2016). Grouped tripeptide composition provides tripeptide information (Tan et al., 2019). CTD provides distribution patterns of a specific structural property for residues (Cheng et al., 2018) and indirectly contains information about 20 amino acid residues, so PseAAC, in our work, does not contain amino acid information.

#### G-Gap Dipeptide Composition

The GGDC proposed by Ding et al. (2014) is the extension of the proximate dipeptide composition, because proteins contain deep correlation of residues relating with hydrogen bonding in secondary structure. For different *g*, the protein sequence *P* with *L* residues is expressed by a 400-dimensional GGDC as follows:

(1) $$P={\left[f_{1}^{g},f_{2}^{g},\ldots ,f_{\varepsilon }^{g},\ldots ,f_{400}^{g}\right]}^{T}$$

where *T* is called the transposing operator, the $f_{\varepsilon }^{g}$ can be calculated by:

(2) $$f_{\varepsilon }^{g}=n_{\varepsilon }^{g}/\left(L-g-1\right)$$

where the $n_{\varepsilon }^{g}$ denotes the absolute occurrence number of the GGDC in a protein. Since previous studies (Ding H. et al., 2016) have shown that *g* = 2 has the best prediction effect, only 2-gap was used in our experiments.

#### Pseudo–Amino Acid Composition

Hydrophobicity, hydrophilicity, and other physicochemical properties are important characteristics of amino acids. In order to incorporate these properties with amino acid composition, two types of PseAAC were used. In our work, motived by PseAAC, the protein sample, can be expressed as follows:

(3) $$\left\{ \begin{array}{l} \tau_{1}\;=\;\frac{1}{L\;-\;1}\;\sum_{i\;=\;1}^{L\;-\;1}\;H_{k,k+1}^{1}\\ \tau_{2}\;=\;\frac{1}{L\;-\;1}\;\sum_{i\;=\;1}^{L\;-\;1}\;H_{k,k+1}^{2}\\ \cdots \\ \tau_{n}\;=\;\frac{1}{L\;-\;1}\;\sum_{i\;=\;1}^{L\;-\;1}\;H_{k,k+1}^{n}\\ \tau_{n+1}\;=\;\frac{1}{L\;-\;2}\;\sum_{i\;=\;1}^{L\;-\;2}\;H_{k,k+2}^{1},\;(l\;<\;L)\\ \tau_{n+2}\;=\;\frac{1}{L\;-\;2}\;\sum_{i\;=\;1}^{L\;-\;2}\;H_{k,k+2}^{2}\\ \cdots \\ \tau_{\lambda n}\;=\;\frac{1}{L\;-\;\lambda }\;\sum_{i\;=\;1}^{L\;-\;\lambda }\;H_{k,k+\lambda }^{n} \end{array}\right.$$

$H_{k,k+\lambda }^{n}$ th residue and the (*k* +λ)-th residue; L is length of sample. After experimental comparison, we selected 10 physical and chemical properties containing hydrophobicity, hydrophilicity, amino acid side chain group mass, -COOH group dissociation constant, -NH3 group dissociation constant, isoelectric point at 25°C, rigidity, flexibility, irreplaceability, and polarity. We used λ = 15.

#### GTPC and CTD

iFeature is a comprehensive Python-based toolkit that contains four major functions: feature representation, dimensionality reduction algorithms, feature selection algorithms, and feature clustering algorithms (Chen Z. et al., 2018). In our study, we have used GTPC and CTD provided by iFeature (Chen Z. et al., 2018) to extract numerical descriptors from samples. Grouped tripeptide composition converts protein sequences into 125-dimensional digital features expressed as follows:

(4) $$f\,\left(r,s,t\right)=\frac{N_{r\,s\,t}}{N-1},r,s\in \left\{g\,1,g\,2,g\,3,g\,4,g\,5\right\}$$

where *N*_*rst*_ denotes the number of tripeptides in groups *r*, *s*, and t (Chen Z. et al., 2018). *N* is the length of a protein.

CTD converts protein sequences into 39-dimensional digital features defined as follows:

(5) $$C\,\left(r\right)=\frac{N_{r}}{N},r\,\left\{p\,o\,l\,a\,r,n\,e\,u\,t\,r\,a\,l,h\,y\,d\,r\,o\,p\,h\,o\,i\,c\right\}$$

where *N*(*r*) represents the number of residue type *r* in the peptide sequence (Chen Z. et al., 2018). Thus, samples are transformed into 164 -dimensional features.

### Support Vector Machine

Support vector machine is a classical machine learning algorithm and has been widely adopted in computational biology (Jiang et al., 2013; Zhao et al., 2015, 2017; Ding H. et al., 2016; Ding et al., 2016a, b; Dao et al., 2018; Feng et al., 2018; Manavalan et al., 2018a, b; Zhang et al., 2018; Chao et al., 2019; Chen et al., 2019a; Wang et al., 2019; Basith et al., 2020). For nonlinear samples, its projects inputted data into high-dimensional spare by a kernel function. There are four kernel functions including Sigmoid function, Gaussian function, line function, and polynomial function, among which Gaussian function is most commonly used. *C* and *g* are the most important parameters to adjust performance of Gaussian function. The value of *g* is related to the partitioning of samples, and the value of *C* determines the tolerance of the model. In our work, SVC functions in Scikit-learn (Swami and Jain, 2012), based on Python, are used to build models, and Gaussian functions are used as kernel functions, because the Gaussian function can efficiently map small samples with fewer features to high-dimensional space and distinguish positive and negative samples with high Ac. In addition, the GridSearchCV function in Scikit-learn was used to optimize the parameters *C* and *g*.

### Feature Selection Method

Because one type of feature does not fully represent the characteristics of a protein sequence, the combination of features is a good approach to perform classifications. The combined features could also cause a lot of inconvenience, such as noise, dimension disaster, and so on. Analysis of variance (Feng et al., 2013; Tang et al., 2017; Xianfang et al., 2019), principal component analysis (Dong et al., 2015), minimal redundancy maximal relevance (Ding et al., 2013), maximum relevance maximum distance (Zou et al., 2016b), and increment of diversity (Zuo and Li, 2009; Zhao et al., 2010; Fan and Li, 2012) can solve these problems. In our study, ANOVA is used to screen the best feature set; the idea is to calculate the ratio of the categories to sample variance. Obviously, features with larger ratios are more suitable for classification. The details can be referred from Feng et al. (2013), Tang et al. (2017) and Xianfang et al. (2019).

### Performance Evaluation

In statistical prediction, the performance of the model needs to be measured by some methods and parameters (Chen et al., 2017, 2019b; Ding et al., 2017; Tang et al., 2018; Yang et al., 2018). The cross-validation test has been widely used to evaluate methods (Yang et al., 2019; Zhu et al., 2019). To provide a fair comparison, we used the jackknife test in this study. The four parameters, namely, sensitivity (Sn), specificity (Sp), Ac, and Matthew correlation coefficient (MCC), are used to evaluate the performance of the model (Liu et al., 2018; Manavalan et al., 2018c, 2019a,c; Basith et al., 2019), which are defined as follows:

(6) $$\left\{ \begin{array}{c} S_{n}=\frac{T\,P}{T\,P+F\,N}\\ S_{p}=\frac{T\,P}{T\,P+F\,N}\\ A_{c}=\frac{T\,P+F\,N}{T\,P+F\,P+T\,N+F\,N}\\ M\,C\,C=\frac{\left(T\,P\times T\,N\right)+\left(F\,P\times F\,N\right)}{\left(T\,P+F\,N\right)\,\left(T\,N+F\,P\right)\,\left(T\,P+F\,P\right)\,\left(T\,N+F\,N\right)} \end{array}\right.$$

where TP and TN are the number of the correctly identified positive samples and the number of the correctly identified negative samples; FP indicates the number of negative samples recognized as positive samples; FN indicates the number of positive samples recognized as negative samples. Also, the area under receiver operating characteristic (ROC) curve (AUC) is often used to evaluate the performance of binary classification models.

## Results

### Discriminating Phage Enzymes From Nonenzymes

**FIGURE 1:**
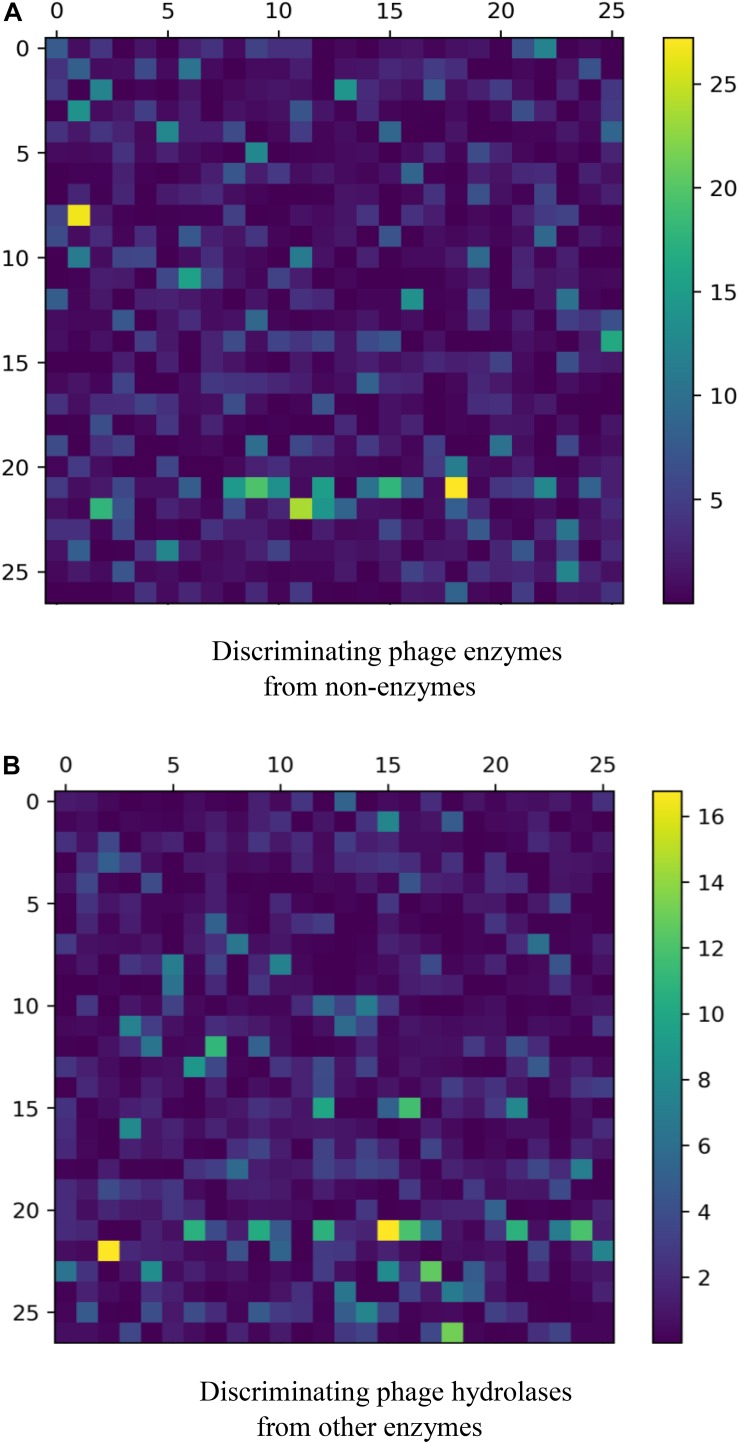
A plot showing the *F*-values for **(A)** discriminating phage enzymes from nonenzymes and **(B)** discriminating phage hydrolases from other enzymes.

For a new sequenced phage protein, we first need to judge whether the phage protein is an enzyme. Thus, the predictive performances of three combined vectors were investigated by using SVM with jackknife test. First, samples are expressed by three kinds of combinations: GGDC combined with PseAAC, GTPC combined with CTD, and all features. Prediction results are listed in Table 1. We observed that all features cannot achieve the best Ac. The reason is maybe noise or redundant information. Thus, we performed feature selection for three feature combinations to discover the best feature subsets. The results are also shown in Table 1. After feature selection, the highest Ac was obtained by using 191 features, which was based on all features. Figure 1A was drawn to show the *F-*value for all features. The above results implied that the information of phage enzymes requires multiple types of feature expressions. However, noises or redundant information may be results in the poor predictive capabilities of other groups, and the combining vectors of the first and second groups cannot fully express the peculiarity of the samples, which lead to its poor prediction effect. Subsequently, we investigated the performance of four classifiers, including random forest (RF), multilayer perceptron (MLP), k-nearest neighbor (KNN), and SVM, whose input features are the third set of 191-D optimal features. The result parameters of four classifiers have been exhibited in Table 2. We found the highest Ac of 85.1% and MCC of 70.3%. The AUC reaches to 89.3% by using SVM. k-Nearest neighbor has achieved the highest Sn of 98% with the lowest Sp of 16%. Moreover, performance of RF has an Sn of 73%, Sp of 76%, Ac of 75.2%, MCC of 0.490, and AUC of 0.798, respectively. Similarity, MLP obtained 77, 84, 81.2, 0.61, and 0.858%, respectively, for Sn, Sp, Ac, MCC, and AUC. These data indicate that SVM is the most suitable for distinguishing phage enzymes.

**TABLE 1:** The results by using different features for phage enzymes prediction.

Combined vector features	Original feature		Optimal features	
	Accuracy	Dimensions	Accuracy	Dimensions
GGDC + PseAAC	74.5%	550	83.1%	154
GTPC + CTD	67.8%	164	77.6%	35
GGDC + PseAAC + GTPC + CTD	72.9%	714	85.1%	191

**GGDC, g-gap dipeptide composition; CTD, composition transition and distribution; PseAAC, pseudo–amino acid composition; GTPC, grouped tripeptide composition.**

**TABLE 2:** The comparison of different classifiers for predicting phage enzymes.

Classifier	Sn	Sp	Ac	MCC	AUC
KNN	0.98	0.16	0.702	0.232	0.664
RF	0.73	0.76	0.752	0.490	0.798
SVM	0.83	0.88	0.851	0.703	0.897
MLP	0.77	0.84	0.812	0.610	0.858

**SVM, support vector machine; RF, random forest; MLP, multilayer perceptron; KNN, k-nearest neighbors; Sn, sensitivity; Sp, specificity; Ac, accuracy; MCC, Matthew correlation coefficient; AUC, area under receiver operating characteristic (ROC) curve.**

### Discriminating Phage Hydrolases From Other Enzymes

When a phage protein is predicted as a phage enzyme, it is necessary to immediately judge whether the enzyme is a hydrolase. Like phage enzyme prediction, the performances of three combined vectors on phage hydrolase prediction were also examined by using SVM with jackknife cross-validation. As shown in Table 3, the three combined vectors were also processed by the feature selection algorithm, which not only improves the Ac but also greatly reduces the dimensions. Obviously, ANOVA can remove redundant information from features. It should be noticed that the optimal features (61-D) obtained from GGDC combined with PseAAC could produce the maximum Ac of 94.3%. This phenomenon indicates that features with a large *F-*value in the second group are not suitable for expressing hydrolases. The heat map for the features is also drawn in Figure 1B. Similarly, we compared the performances of different classifiers. In Table 4, KNN has yielded Ac of 81.4%, whereas KNN has obtained Ac of 84.64%. The performance of MLP is 93% Sn, 91% Sp, 92.7% Ac, 0.837 MCC, and 0.948 AUC. Support vector machine with Radial Basis Function (RBF) as kernel function gained the best prediction performance (94.3% Ac).

**TABLE 3:** The results by using different feature for discriminating phage hydrolases from other enzymes.

Combined vector features	Original features		Optimal features	
	Accuracy	Dimensions	Accuracy	Dimensions
GGDC + PseAAC	75.8	550	94.3%	61
GTPC + CTD	76.6%	164	86.4%	37
GGDC + PseAAC + GTPC + CTD	75.8%	714	92.7%	89

**GGDC, g-gap dipeptide composition; CTD, composition transition and distribution; PseAAC, pseudo–amino acid composition; GTPC, grouped tripeptide composition.**

**TABLE 4:** The comparison of different classifiers for discriminating phage hydrolases from other enzymes.

Classifier	Sn	Sp	Ac	MCC	AUC
KNN	0.70	0.89	0.814	0.588	0.863
RF	0.91	0.80	0.86	0.722	0.898
SVM	0.96	0.93	0.943	0.886	0.961
MLP	0.93	0.91	0.927	0.837	0.948

**SVM, support vector machine; RF, random forest; MLP, multilayer perceptron; KNN, k-nearest neighbors; Sn, sensitivity; Sp, specificity; Ac, accuracy; MCC, Matthew correlation coefficient; AUC, area under receiver operating characteristic (ROC) curve.**

### Performance Comparison With Existing Methods

In order to prove that our proposed model performs better than the model by Ding H. et al. (2016), who first used computational methods to predict hydrolases, the performance indexes of the two models were recorded in Table 5. In discriminating phage enzymes from nonenzymes, our model is better in Ac and Sp that are 85.1 and 88.0%, respectively. In discriminating phage hydrolases from other enzymes, all the evaluated indexes of our proposed model are better than those of Ding H. et al. (2016). Indeed, hydrolyzing enzymes adopt two types of features to encode samples. Compared with Ding and colleagues’ experiment, we have selected more kinds of features in the sample expression, which makes the digital features of the sample more informative.

**TABLE 5:** Comparison of predictive performance with exist method.

		Ac	Sp	Sn
Discriminating phage enzymes from nonenzymes	(Ding H. et al., 2016)	84.3%	81.7%	87.1%
	This study	85.1%	88.0%	83.0%
Discriminating phage hydrolases from other enzymes	(Ding H. et al., 2016)	93.5%	92.8%	94.5%
	This study	94.3%	93.0%	96.0%

**Sn, sensitivity; Sp, specificity; Ac, accuracy.**

## Discussion

The purpose of this study is to establish a predictive model to predict phage enzymes and hydrolases. In fact, similarity search could be used to perform sequence analysis and function prediction. However, the strategy cannot work well on low-similar sequences. Especially, the phage genes display the extreme diversity. Protein functions are inextricably linked to correlation of nucleotides or residues, physicochemical properties, spatial structure, and other information. Therefore, we used multiple characteristics to represent phage and hydrolase, but this method has some problems that multiple features contain too much redundant information; different types of features are suitable for different samples. On the basis of the feature selection technique, promising results for phage enzymes and hydrolases prediction were achieved. In the future, we will pay more attention on deep learning, which has solved several protein prediction problems (Peng et al., 2018; Wei et al., 2018a, 2019; Yu et al., 2018; Lv et al., 2019) and may get well performance on this topic. Moreover, we will establish a free webserver that facilitates users to download data and predict phage hydrolases.

## Data Availability Statement

The datasets generated for this study can be found in the http://lin-group.cn/server/PHYPred.

## Author Contributions

H-FL and HT designed the study. H-FL carried out all data collection and drafted the manuscript. X-FW and HT revised the manuscript. All authors approved the final manuscript.

## Conflict of Interest

The authors declare that the research was conducted in the absence of any commercial or financial relationships that could be construed as a potential conflict of interest.

## Abbreviations

CTD: composition transition and distribution
SVM: support vector machine
RF: random forest
MLP: multilayer perceptron
KNN: k-nearest neighbors
Sn: sensitivity
Sp: specificity
Ac: accuracy
MCC: matthew correlation coefficient
PseAAC: pseudo–amino acid composition
GTPC: grouped tripeptide composition
ROC: receiver operating characteristic
AUC: area under receiver operating characteristic (ROC) curve
GGDC: g-gap dipeptide composition
ANOVA: analysis of variance
RBF: Radial Basis Function
ORFs: Open Reading Frames.
